# Relationship Between the Plasma Concentration of C-Reactive Protein and Severity of Peripheral Arterial Disease

**DOI:** 10.4137/cmc.s1062

**Published:** 2008-12-23

**Authors:** Joaquin De Haro, Francisco Acin, Francisco Jose Medina, Alfonso Lopez-Quintana, Jose Ramon March

**Affiliations:** Department of Angiology and Vascular Surgery, Getafe University Hospital.

**Keywords:** C-reactive protein, peripheral arterial disease, inflammatory origen

## Abstract

**Objective:**

To determine whether the increase in plasma levels of C-Reactive Protein (CRP), a non-specific reactant in the acute-phase of systemic inflammation, is associated with clinical severity of peripheral arterial disease (PAD).

**Methods and Results:**

This is a cross-sectional study at a referral hospital center of institutional practice in Madrid, Spain. A stratified random sampling was done over a population of 3370 patients with symptomatic PAD from the outpatient vascular laboratory database in 2007 in the order of their clinical severity: the first group of patients with mild chronological clinical severity who did not require surgical revascularization, the second group consisted of patients with moderate clinical severity who had only undergone only one surgical revascularization procedure and the third group consisted of patients who were severely affected and had undergone two or more surgical revascularization procedures of the lower extremities in different areas or needed late re-interventions. The Neyman affixation was used to calculate the sample size with a fixed relative error of 0.1. A homogeneity analysis between groups and a unifactorial analysis of comparison of medians for CRP was done. The groups were homogeneous for age, smoking status, Arterial Hypertension HTA, diabetes mellitus, dyslipemia, homocysteinemia and specific markers of inflammation. In the unifactorial analysis of multiple comparisons of medians according to Scheffé, it was observed that the median values of CRP plasma levels were increased in association with higher clinical severity of PAD (3.81 mg/L [2.14–5.48] vs. 8.33 [4.38–9.19] vs. 12.83 [9.5–14.16]; p < 0.05) as a unique factor of tested ones.

**Conclusion:**

Plasma levels of CRP are associated with not only the presence of atherosclerosis but also with its chronological clinical severity.

## Introduction

Laboratory and experimental evidence have shown that inflammatory processes play a central role in the development, progression and outcomes of atherosclerosis. Several studies suggest that patients at high risk of developing atherothrombotic disease suffer from chronic systemic inflammation.^1^ More specifically, increased levels of C-Reactive Protein (CRP), a seric marker of systemic inflammation, have been associated with a higher risk of: acute myocardial infarction or sudden death in patients diagnosed with either stable or unstable angina,^2^ symptomatic ischemic coronary disease in elderly patients,^3^ death caused by coronary disease^4^ and first episodes of myocardial infarction or cerebral ischemic stroke in otherwise apparently healthy people.^1^ Likewise, some hypotheses propose that the seric concentration of CRP could have a predictive value in preclinical atherosclerosis as a molecular indicator of the severity of the disease. Indeed, recent research concludes that high-sensitivity measurements of CRP provide an adjunctive method for global assessment of cardiovascular risk, in addition to traditional screening methods.^5^ Thus, CRP plasma levels would add information of prognostic value to that obtained from plasma lipid levels based on Framingham study-based criteria.^6^

Peripheral Arterial Disease (PAD) includes all the clinical entities resulting from arterial blood flow obstruction, excluding coronary and intracranial vascular beds. In this paper, the focus is on PAD affecting just the lower extremities.

Currently, there are few data relating CRP levels to PAD. Evidence^7^ is found suggesting that increased basal levels of CRP in otherwise healthy people are associated with a higher risk of intermittent claudication and peripheral surgical revascularization.

## Objective

The aim of this study is to determine whether the increase in CRP concentration, a non-specific reactant in the acute-phase of systemic inflammation that can be easily and reliably measured, is associated with clinical severity of PAD.

## Material and Methods

A prospective cross-sectional study was designed in order to find out a possible relationship between CRP plasma levels and the clinical severity of PAD in a target population of patients with PAD, in the south of Madrid in Spain with a population of 3 million. For this purpose, a random sample of the target population was used and they were classified as described below, which allowed a prospective assessment of the inflammation markers in a well-established gradation according to the aggression of the disease at the time of presentation.

The study population was made up of 3370 patients diagnosed with PAD registered in the outpatient vascular laboratory database of this hospital. All patients were documented with, at least, a hemodynamic study with arterial Doppler of the lower extremities that demonstrated an ankle-brachial index lower than 0, 8.

With the purpose of evaluating the association between CRP plasma levels and the clinical form of PAD developed, three patient groups were formed as follows:

- **Group 1**: patients with symptomatic PAD in the lower extremities who did not require surgical revascularization
- **Group 2**: patients with symptomatic PAD in the lower extremities who had undergone only one surgical revascularization procedure (either open or endovascular surgery) of the lower extremities (in the aorto-iliac or infra-inguinal area) before December 31st 2005
  ○ (A latent period of 12 months without an operation is considered to be a sign of no progression of the disease).
- **Group 3**: patients with symptomatic PAD in the lower extremities who had undergone two or more surgical revascularization procedures (either open or endovascular surgery) of the lower extremities (in the aorto-iliac or infra-inguinal area) in different areas or patients with symptomatic PAD in the lower extremities who needed another procedure in the same area with a delay of more than 12 months between procedures
- (The aorto-iliac area is considered to be different from the infra-inguinal area, and this last one also considered to be a different area from that of the lower limbs).

The total population of 3370 patients were divided according to their clinical presentation into the three above-mentioned groups resulting in 2900, 380 and 90 patients respectively.

The Neyman’s affixation was used to calculate the sample size needed for each stratus (N1, N2, N3).^8^ For a fixed relative error of 0.1 calculated for the average, the sample size had to be 51 patients. The affixations were then calculated according to Neyman to calculate the size of each stratus: N1 = 23; N2 = 17; N3 = 10. Therefore, 330 patients were randomly chosen from the different groups: 150 from the first group, 130 from the second and 50 from the third. All the patients who had had any infectious disease or other acute illness such as acute coronary or limb ischemia, and those who had undergone any surgical procedure within a 16-week period were excluded from the study, in order to control any possible bias by elevated CRP levels resulting due to these processes. Any patient with foot ulcers was also excluded.

Blood samples were taken from all of them, followed by standard blood processing and storing procedures. The plasma CRP levels along with the plasma lipidic profile (total cholesterol levels, triglycerides and HDL, LDL and VLDL lipoproteins), the plasma homocystein concentration, serum creatinine level and the glycosylated hemoglobin level of each patient were determined. A complete blood count was done. Clinical history about the cardiovascular risk factors, associated medication and symptoms of ischemia were obtained from the patients through individual interviews. At the time of blood sampling, all patients were in a Rutherford category of three or minor.

The CRP plasma concentrations were determined by an automatic ultrasensitive immunoassay (Roche Diagnostics GMBH) with a lower-limit detection of 0.2 mg/L and a coefficient of variation of 4.2% in 4 mg/L and 6.3% in 1 mg/L.

The CRP plasma concentration median within each group was calculated along with the statistical significance in the differences found between them by means of stratified unifactorial analysis of multiple comparisons of medians according to Schaffé, who uses the F Distribution of Snedecor, which is not influenced by a lack of homogeneity within the groups.^8^

The homogeneity within the groups was checked regarding CRP plasma levels and the following variables: age, smoking status, hypertension, diabetes, hyperlipidemia and lipidic plasma profile, COPD, coronary heart disease, cerebrovascular disease, homocysteinemia, serum creatinine levels, LDH plasma levels, and leukocytes, neutrophils, lymphocytes and reticulocytes.

A multivariant analysis among groups was also performed to rule out possible confounding factors for the following variables: age, smoking status, hypertension, diabetes, hyperlipidemia and lipidic plasma profile, COPD, coronary heart disease, cerebrovascular disease, homocysteinemia, serum creatinine levels, blood markers of acute inflammation (leukocytosis, neutrophilia, basophilia, eosinophilia, monophilia, percentage of reticulocytes) and associated anti-inflammatory medication (aspirin and trifusal prescribed for PAD and steroid and non-steroid anti-inflammatory drugs prescribed for other diseases).

All the variable data were expressed as median ± standard deviation except the variable CRP, expressed as median [quartil25–quartil75].

A p-value > 0.05 was considered to be significant.

## Results

Table 1 displays the univariate analysis of clinical and analytical characteristics that are currently known as being associated with PAD. No significant differences were found in any of the variables studied (age, cardiovascular risk factors such as smoking, hypertension, diabetes, dyslipemia, COPD, plasma homocystein levels, lipidic profile or serum creatinine levels—a renal failure marker) that could act as confounding factors in the study of the association between CRP plasma levels and clinical severity of PAD.

No significant difference was found in the composition of the groups relating to the existence of atherosclerotic disease in the coronary and cerebral vascular beds.

These results also show homogeneity between the groups relating to cardiovascular risk factors.

The multivariate analysis made on the factors associated with PAD stages resulted in no significant differences for any variable.

Table 2 displays plasma levels of specific markers of inflammation and blood counts in each clinical group at the time of plasma CRP level testing. There were no differences between groups for any tested marker and the levels show that there was no acute inflammatory process at the time of blood sampling that could have affected the study.

No difference was found between groups concerning associated medication that could have affected CRP plasma levels: statins, ACE-inhibitors, angiotensin receptor blockers and beta blockers.

Intragroup homogeneity test applied to CRP plasma levels and all the variables obtained from multivariate analysis among the groups show homogeneity in each group for all variables.

Once the intragroup homogeneity was tested the data of CRP plasma levels were analysed (Table 3).

In the unifactorial analysis of multiple comparisons of medians according to Scheffé for CRP plasma levels, it was found that in each group, an F statistic value of 24.113 was obtained which showed a significant grade much lower than 0.05 (ca. 0.000), thereby demonstrating a statistically significant difference between CRP plasma levels of the patients grouped according to the clinical severity of their PAD. This suggests, therefore, the existence of a consistent relationship between both variables.

**Figure 1.**
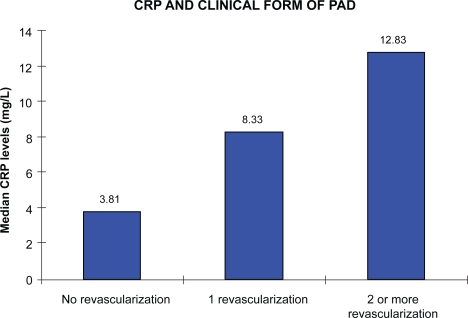
Association tendency between crp and clinical severity of PAD.

As it can seen, median values of CRP plasma levels were increased in association with higher clinical severity of PAD (3.81 mg/L; 8.33 mg/L; 12.83 mg/L) (Figure 1). These data indicate a direct relationship between CRP plasma levels and clinical severity of PAD.

## Discussion

The data presented here allows the inference that there exists an association in patients with symptomatic PAD between their CRP plasma levels and the way the disease develops clinically. In previous studies the CRP basal levels in apparently healthy patients have already been linked to the risk of developing symptomatic PAD.^7^ Therefore, the results obtained here allow widening of the previously acknowledged role of CRP as a marker of vascular risk, not only in the coronary and cerebral beds but also in the peripheral circulation, associating it directly with the severity and extension of PAD.

From a more integrative perspective based upon the results of this study and those of previous studies,^1^–^4^,^7^ it may be hypothesized that CRP could serve as a molecular marker of the presence and clinical severity of systemic atherosclerotic disease.

Studies trying to prove the role of CRP in cardiovascular risk assessment use “high-sensitive” CRP measurements since standard clinical assays for CRP typically have a lower detection limit of 1 to 3 mg/L. Thus, these assays lack sensitivity within the low-normal range and cannot be used effectively for vascular risk prediction. In recognition of this limitation, these epidemiological studies use “high-sensitivity” tests to determine CRP levels with excellent fidelity and reproducibility across the normal range.^9^ In inflammatory status, as in the symptomatic PAD that this study aims to prove, CRP levels are increased significantly and thus standard CRP assays help in accurate measurement of the severity of the disease.

Non-specific inflammatory markers increase in acute infection or trauma and in individuals with clinically apparent inflammatory conditions such as rheumatoid arthritis or lupus. All the patients who had had any infectious disease or other acute illness such as acute coronary or limb ischemia within a 16-week period, and those suffering from chronic inflammatory disease were excluded from the study. It was also ensured that the specific acute-phase inflammatory cells and serum markers were similar between the three groups.

The mechanism of CRP’s association with an increase in the risk and severity of atherosclerotic disease is only suggested by diverse hypotheses. It is believed that CRP, in addition to being a hepatic marker of systemic inflammation, could have a procoagulant effect associated with its capacity to promote the expression of tissue factor.^10^

Experimental studies suggest that CRP can induce activation of the complement system,^11^ bind to human neutrophils,^12^ be found in the endothelium of the blood vessels^13^ and can be probably involved in the secretion of cellular adhesion molecules that play an important role in the leukocyte adhesion and migration across the vascular wall—a very important step in the beginning of atherosclerosis.^14^

On the other hand, there are also hypotheses that suggest that low-grade inflammation detected by CRP indicates a chronic infection process. Currently, there are cross-sectional and case-control studies that have reported elevated antibody titers against *Helicobacter pylori, Chlamydia pneumoniae* and Cytomegalovirus in patients with a high prevalence of heart disease.^15^ It is also possible that the association between CRP and atherosclerotic disease is a product of cytokines such as interleukin-6 (IL-6) that promote leukocyte adhesion and stimulate CRP production. Studies have demonstrated the relationship between inflammation and endothelial dysfunction and thereby the deregulation in nitric oxide metabolism.^16^,^17^

Although previous studies have shown positive associations between ankle-brachial index and measurements of hemostatic and inflammatory markers,^18^ there have been no known previous studies that have directly compared the relationship between CRP plasma levels in patients with symptomatic PAD and the severity in the chronological behaviour of the disease.

This study has some limitations as it is cross-sectional and thereby there is a difficulty in determining whether the higher CRP levels are causally related to the clinical severity of PAD. However, this relationship reinforces the important role of the inflammatory pathway in the form of presentation of PAD in the symptomatic states and not just in the case of underlying asymptomatic disease, where CRP is well-known as a predictor and marker of developing peripheral vascular disease.^19^–^24^ The second limitation is that the long-term effects of the surgical procedures, as could be in case of the inflammatory changes at anastomotic sites, would be able to elevate the CRP plasma levels. Experimental *in vivo* and pathologic studies have shown that inflammatory reaction in the atherosclerotic plaque is exponentially greater than that produced in the anastomosis by the vascular wall trauma.

In conclusion, plasma levels of CRP are associated not only with the presence of atherosclerosis but also with its clinical severity in the target population of this study. For universality of this conclusion more studies are required with a larger target population.

## Figures and Tables

**Table 1.** Cardiovascular risk factors.

	**Patients with no surgical revascularization (Group 1)**	**Patients with only 1 revascularization (Group 2)**	**Patients with 2 or more revascularizations (Group 3)**	**p-value**
Sample size	150	130	50	
Age (mean)	66.87 ± 2.1	62.41 ± 3.1	66.91 ± 1.9	0.443
Smoking (%)	26.08	35.29	45.45	0.263
HTA (%)	65.21	70.58	81.81	0.341
Diabetes (%)	60.86	17.64	36.36	0.068
Dyslipemia (%)	52.17	58.82	27.27	0.273
COPD (%)	13.04	11.76	9.09	0.750
Coronary disease (%)	30.43	47.05	36.36	0.595
Cerebrovascular disease (%)	8.69	11.76	9.09	0.917
Homocysteinemia (μmol/L)	10.88 ± 1.1	10.10 ± 0.8	10.02 ± 0.9	0.853
Total cholesterolemia (mg/dL)	197.39 ± 11.6	204 ± 19	185.36 ± 2101	0.864
HDL (mg/dL)	56.17 ± 7.1	58 ± 5.9	46.64 ± 6.8	0.063
LDL (mg/dL)	120.30 ± 14	112.76 ± 13.9	106.73 ± 10.3	0.652
VLDL (mg/dL)	23.43 ± 5.7	31.82 ± 5	33.91 ± 6.3	0.390
Trigliceridemia (mg/dL)	123.61 ± 11.9	146.53 ± 13	158.91 ± 14.3	0.488
Creatininemia (mg/dL)	1.05 ± 0.1	0.93 ± 0.08	1.45 ± 0.1	0.523
HbA1c (%)	6.51	6.04	5.87	0.571

**Table 2.** Specific markers of inflammation.

	**Patients with no surgical revascularization (Group 1)**	**Patients with only 1 revascularization (Group 2)**	**Patients with 2 or more revascularizations (Group 3)**	**p-value**
Leucocytes (10^3^/μl)	7.663 ± 1.1	8.373 ± 1.3	7.247 ± 1.2	0.100
Neutrophils (10^3^/μl)	4.646 ± 0.9	4.691 ± 0.8	4.281 ± 0.4	0.519
Lymphocytes (10^3^/μl)	1.992 ± 0.25	2.642 ± 0.8	1.762 ± 0.9	0.796
Monocytes (10^3^/μl)	0.582 ± 0.2	0.577 ± 0.1	0.396 ± 0.15	0.190
Eosinophils (10^3^/μl)	0.296 ± 0.11	0.272 ± 0.11	0.478 ± 0.13	0.884
Basophils (10^3^/μl)	4.043E-02 ± 0.26	4.529E-02 ± 1.2	3.818E-02 ± 1.9	0.234
Reticulocytes (%)	1.114	1.244	1.467	0.216
LDH (U/L)	325.83 ± 22	330.53 ± 32	365.18 ± 41	0.106

No statistical differences for major cardiovascular risk factors between groups.

**Table 3.** Clinical severity of peripheral arterial disease according to C-reactive proteinplasma levels.

	**Size sample**	**CRP median concentration**	**Quartil25–Quartil75**	**Range**
Group 1	150	3.81	2.14–5.48	0.12–12.1
Group 2	130	8.33	4.38–9.19	0.83–16.5
Group 3	50	12.83	9.5–14.16	4.67–19.8
